# Coumarin-chalcone derivatives as dual NLRP1 and NLRP3 inflammasome inhibitors targeting oxidative stress and inflammation in neurotoxin-induced HMC3 and BE(2)-M17 cell models of Parkinson's disease

**DOI:** 10.3389/fnagi.2024.1437138

**Published:** 2024-10-01

**Authors:** Te-Hsien Lin, Ya-Jen Chiu, Chih-Hsin Lin, Yi-Ru Chen, Wenwei Lin, Yih-Ru Wu, Kuo-Hsuan Chang, Chiung-Mei Chen, Guey-Jen Lee-Chen

**Affiliations:** ^1^Department of Neurology, Chang Gung Memorial Hospital, Chang Gung University School of Medicine, Taoyuan, Taiwan; ^2^Department of Life Science, National Taiwan Normal University, Taipei, Taiwan; ^3^Department of Chemistry, National Taiwan Normal University, Taipei, Taiwan

**Keywords:** Parkinson's disease, therapeutics, neuroinflammation, oxidative stress, MPP^+^, human HMC3 and BE(2)-M17 cells

## Abstract

**Background:**

In Parkinson's disease (PD) brains, microglia are activated to release inflammatory factors to induce the production of reactive oxygen species (ROS) in neuron, and vice versa. Moreover, neuroinflammation and its synergistic interaction with oxidative stress contribute to the pathogenesis of PD.

**Methods:**

In this study, we investigated whether in-house synthetic coumarin-chalcone derivatives protect human microglia HMC3 and neuroblastoma BE(2)-M17 cells against 1-methyl-4-phenyl pyridinium (MPP^+^)-induced neuroinflammation and associated neuronal damage.

**Results:**

Treatment with MPP^+^ decreased cell viability as well as increased the release of inflammatory mediators including cytokines and nitric oxide in culture medium, and enhanced expression of microglial activation markers CD68 and MHCII in HMC3 cells. The protein levels of NLRP3, CASP1, iNOS, IL-1β, IL-6, and TNF-α were also increased in MPP^+^-stimulated HMC3 cells. Among the four tested compounds, LM-016, LM-021, and LM-036 at 10 μM counteracted the inflammatory action of MPP^+^ in HMC3 cells. In addition, LM-021 and LM-036 increased cell viability, reduced lactate dehydrogenase release, ameliorated cellular ROS production, decreased caspase-1, caspase-3 and caspase-6 activities, and promoted neurite outgrowth in MPP^+^-treated BE(2)-M17 cells. These protective effects were mediated by down-regulating inflammatory NLRP1, IL-1β, IL-6, and TNF-α, as well as up-regulating antioxidative NRF2, NQO1, GCLC, and PGC-1α, and neuroprotective CREB, BDNF, and BCL2.

**Conclusion:**

The study results strengthen the involvement of neuroinflammation and oxidative stress in PD pathogenic mechanisms, and indicate the potential use of LM-021 and LM-036 as dual inflammasome inhibitors in treating both NLRP1- and NLRP3-associated PD.

## 1 Introduction

Parkinson's disease (PD), characterized by resting tremor, rigidity, bradykinesia, and postural instability, is the second most common neurodegenerative disorder among the elderly (Jankovic, 2008). The pathological studies reveal a massive loss of dopaminergic neurons located in the pars compacta of the substantia nigra (SN) of the midbrain and subsequent depletion of dopamine in their projections (Surmeier et al., 2017). The neurodegeneration of PD could be caused by a complex interaction of genetic and environmental factors (Kalia and Lang, 2015). Owing to the discovery of causative PD genes, several pathways have been identified and attributed to neurodegeneration, including abnormal α-synuclein protein loads, mitochondrial abnormalities and increased oxidative stress, neuroinflammation, ubiquitin-proteasome dysfunction, impaired autophagolysosome and mitophagy, deficient synaptic exocytosis and endocytosis, and altered endosomal-lysosomal trafficking (Morris et al., 2024).

Neuroinflammation has emerged as an important mechanism contributes to pathogenesis of PD (Tansey et al., 2022) and anti-inflammatory drugs have shown protective effects in both animal models and epidemiological studies (Bassani et al., 2015). Inflammasomes are considered to be the key players in inflammation-mediated neurodegenerative diseases (Singh et al., 2023). Among them, NOD-like receptor (NLR) family pyrin domain containing protein 3 (NLRP3) is mainly activated in microglia, whereas NLR family pyrin domain containing protein 1 (NLRP1) in neurons (Kummer et al., 2007; Kaushal et al., 2015). When NLRs sense pathogen-associated molecular patterns (PAMPs) as well as the host-derived signals known as damage-associated molecular patterns (DAMPs) in the progression of neurodegeneration, they induce the assembly of a large complex called inflammasomes, which lead to subsequent maturation of pro-inflammatory cytokines interleukin (IL)-1β and IL-18 causing further inflammatory damage to cells (Lin and Mei, 2020). Substantial evidence has shown α-synuclein aggregates may activate NLRP3 inflammasomes and aggravate neurodegeneration in PD (Nguyen et al., 2022).

Mitochondrial dysfunction and increased oxidative stress also significantly mediate the pathogenesis of PD (Prasuhn et al., 2021). In response to oxidative stress or reactive oxygen species (ROS), the transcription factor nuclear factor-erythroid 2-related factor 2 (NRF2), a master regulator of cellular defense, is activated to enhance the expression of antioxidants such as NAD(P)H quinone oxidoreductase 1 (NQO1), heme oxygenase 1 (HO-1), glutamate-cysteine ligase catalytic subunit (GCLC), and glutathione S-transferase pi 1 (GSTP1) (Calkins et al., 2009). Besides, activated NRF2 also upregulates the expression of peroxisome proliferator-activated receptor-γ coactivator 1-α (PGC-1α) (Athale et al., 2012), a key metabolic regulator playing important roles in maintaining mitochondrial function and neuronal survival (Panes et al., 2022). Given that ROS can activate NLRP3, NRF2 also plays an important role in regulating NLRP3 by reducing oxidative stress (Rajan et al., 2023). The main sources of ROS are the dysfunctional mitochondria. Several lines of research have shown that mitochondrial dysfunctions including ROS, oxidized mitochondrial DNA, disrupted mitophagy, and mitochondrial fission can induce NLRP3 inflammasome in PD (Khot et al., 2022; Han and Le, 2023). Given that there is a strong link between NLRP3, NRF2, and mitochondrial dysfunction, agents targeting either NLRP3 and/or NRF2 may have additive beneficial effects of anti-inflammation and enhancing mitochondrial biogenesis in PD (Khot et al., 2022; Han and Le, 2023; Rajan et al., 2023). For example, carvacrol provides neuroprotective effects against rotenone-induced neurotoxicity through suppressing NLRP3 and enhancing NRF2 in PD mice model (Shah et al., 2024). Phloretin protects rotenone-induced PD mice against neurotoxicity through modulating NRF2/sequestosome 1 (p62) mediated autophagy and oxidative stress (Shirgadwar et al., 2023). Dimethyl fumarate, an NRF2 activator, displays neuroprotective effects through NRF2/BCL2 interacting protein 3 (BNIP3)/PTEN induced kinase 1 (PINK1) axis in the 1-methyl-4-phenyl pyridinium (MPP^+^)-induced PD mouse model (Pinjala et al., 2024).

Clustering of activated microglia around scattered neurons has been shown in postmortem brains from persons exposed to 1-methyl-4-phenyl-1,2,3,6-tetrahydropyridine (MPTP) (Langston et al., 1999). MPP^+^-induced cell and MPTP-induced mouse models have been used to study the pathogenesis and test the potential therapeutics of PD. These models have shown that activated microglia, NLRP3, and increased proinflammatory cytokines (Zhao et al., 2007; Bournival et al., 2012; Sanchez-Guajardo et al., 2013; Yao et al., 2019) as well as increased oxidative stress (Hare et al., 2013; Mustapha and Mat Taib, 2021), all of which lead to neurodegeneration in PD. Therefore, in this study we would apply MPP^+^-induced neuroinflammation and neurotoxicity models to test the effects of the coumarin-chalcone derivatives, synthesized by our collaborator laboratory.

For the past years, our group has focused on screening novel synthetic compounds to test the therapeutic effects on several neurodegenerative disease models. Among them, coumarin-chalcone derivative LM-021 (C_20_H_17_NO_4_) works as a CREB enhancer to reduce Aβ and Tau aggregation and provide neuroprotection (Chiu et al., 2021). In addition, LM-021 displayed anti-inflammatory activity in lipopolysaccharides/interferon-γ-stimulated BV-2 microglia and inflammatory cytokine-primed human spinocerebellar ataxia type 3 SH-SY5Y cells (Chen et al., 2024). In the present study, we investigated the neuroprotective potential of LM-021 and related in-house coumarin-chalcone hybrids LM-009 (C_19_H_14_O_5_), LM-016 (C_19_H_14_O_4_) and LM-036 (C_18_H_12_O_5_) in MPP^+^-activated human microglia HMC3 and neuroblastoma BE(2)-M17 cell models of PD.

## 2 Materials and methods

### 2.1 Test compounds and MPP^+^

Four coumarin-chalcone derivative compounds, LM-009, LM-016, LM-021, and LM-036, were synthesized as previously described and examined by NMR spectroscopy (Jang et al., 2012; Lee et al., 2015). LM-036 was named ZN015 or ZN-015 in previous reports (Huang C. C. et al., 2021; Weng et al., 2023). These LM compounds stayed soluble in cell culture medium at concentrations up to 100 μM. Quercetin, positive control for antioxidant assay (Boots et al., 2008), was purchased from Sigma-Aldrich Co. (St. Louis, MO, USA). In addition, complex I inhibitor MPP^+^ was obtained from Cayman Chemical (Ann Arbor, MI, USA).

### 2.2 Bioavailability and blood–brain barrier permeation prediction of LM compounds

Molecular weight (MW), hydrogen bond donor (HBD), hydrogen bond acceptor (HBA), octanol-water partition coefficient (cLogP), and polar surface area (PSA) of LM compounds were analyzed and calculated using ChemDraw (http://www.perkinelmer.com/tw/category/chemdraw). Oral bioavailability and blood–brain barrier (BBB) penetration were predicted based on Lipinski's criteria (MW ≤ 450, HBD ≤ 5, HBA ≤ 10, cLogP ≤ 5) (Lipinski et al., 2001) and PSA (<90 Å^2^) (Hitchcock and Pennington, 2006). In addition, prediction of BBB permeation was performed using online BBB predictor (threshold 0.02; https://www.cbligand.org/BBB/) (Liu et al., 2014).

### 2.3 Cell culture and cytotoxicity assay

Human HMC3 microglial (CRL-3304) and neuroblastoma BE(2)-M17 (CRL-2267) cells were obtained from American Type Culture Collection. BE(2)-M17 develops a better dopaminergic phenotype than SH-SY5Y when treated with retinoic acid to direct neuronal differentiation (Carvajal-Oliveros et al., 2022). Cells were routinely maintained in Dulbecco's Modified Eagle Medium-Nutrient Mixture F12 (DMEM-F12) supplemented with 10% fetal bovine serum (FBS; Thermo Fisher Scientific, Waltham, MA, USA) in a 37°C incubator, with 95% relative humidity and 5% CO_2_ (NuAire, Plymouth, MN, USA).

To evaluate cytotoxicity of LM compounds and MPP^+^, HMC3 (1 × 10^4^) or BE(2)-M17 (2 × 10^4^) cells were plated on 96-well dishes, grown for 20 h, and treated with LM compounds (1–100 μM) or MPP^+^ [HMC3: 0–8 mM; BE(2)-M17: 0–5 mM]. Next day, 20 μl of tetrazolium dye 3-(4,5-dimethylthiazol-2-yl)-2,5-diphenyltetrazolium bromide (MTT, 5 mg/ml; Sigma-Aldrich) was added to the cells at 37°C for 3 h. The resulted insoluble purple formazan products were dissolved by lysis buffer (composition: 10% Triton X-100, 0.1 N HCl, 18% isopropanol). The absorbance of the dissolved product at optical density (OD) 570 nm was read by using a FLx800 fluorescence microplate spectrophotometer (Bio-Tek, Winooski, VT, USA). The half maximal inhibitory concentration (IC_50_) of LM compounds was determined through the construction of a dose-response curve. MPP^+^ at 4 mM (HMC3 cell viability: 65%−69%) or 0.62 mM [BE(2)-M17 cell viability: 79%−84%] was selected to induce inflammation for further experiments.

### 2.4 Antioxidant assays

Stable 1,1-diphenyl-2-picrylhydrazyl (DPPH) radical in ethanol (100 μM; Sigma-Aldrich) was used to measure the antioxidant activity of these LM compounds. After addition of quercetin (positive control) or LM compound (10–160 μM), the mixture was vortexed for 15 s and incubated at room temperature for 30 min. Subsequently, the mixture was measured spectrophotometrically at 517 nm (Multiskan GO microplate spectrophotometer; Thermo Fisher Scientific). The free radical scavenging activity was calculated as the percentage of DPPH discoloration using the formula 1 – (absorbance of sample/absorbance of control) × 100%, with half maximal effective concentration (EC_50_) calculated using the interpolation method.

The test of oxygen radical antioxidant capacity (Ou et al., 2001) was performed using OxiSelect™ kit (Cell Biolabs, San Diego, CA, USA). Briefly, trolox standards (2.5–50 μM) and LM compounds (4–100 μM) were prepared using a serial dilution in a mixture containing 50% acetone and 50% water. After adding fluorescein to blank (50% acetone/50% water), standards, or samples, the mixture was mixed and incubated at 37°C for 30 min. Subsequently, free radical initiator 2,2′-azobis(2-methylpropionamidine) dihydrochloride (AAPH) was added to generate peroxyl radicals (ROO•). The quenching of fluorescent probe over time was recorded at 480 nm excitation and 520 nm emission for 60 min (Bio-Tek FLx800). To quantify the oxygen radical absorbance capacity, the area under the curve (AUC) for blank, standard and samples were calculated. After subtraction of the blank, trolox equivalent (μM) of samples were determined using a calibration curve with trolox standards.

### 2.5 Inflammatory mediator detection in MPP^+^-activated HMC3 cells

To examine anti-inflammatory potential of LM compounds, HMC3 cells were plated into six-well (2 × 10^5^/well) dishes, grown for 20 h, and pre-treated with compounds (10 μM) for 8 h before 4 mM MPP^+^ addition for 20 h. The cell viability was evaluated by MTT assay as described, and the release of nitric oxide (NO) in cell culture medium was examined by Griess reagent kit (Thermo Fisher Scientific) based on a classic protocol.

In addition, HMC3 cells with 10 μM compound treatment were fixed with 4% paraformaldehyde (PFA) for 30 min, permeabilized with 0.1% Triton X-100 for 10 min, and blocked with 2% bovine serum albumin (BSA) for 20 min. Immunocytochemistry staining of HMC3 cells was performed using primary anti-CD68 (CD68 molecule; #76437; 1:1,000; Cell Signaling, Danvers, MA, USA) or anti-MHCII (major histocompatibility complex II; #MS-133-P0; 1:1,000; Thermo Fisher Scientific) antibody at 4°C overnight, followed by Alexa Fluor 555-donkey anti-rabbit (#A-31572; for detecting anti-CD68 antibody) or Alexa Fluor 488-donkey anti-mouse (#A-21202; for detecting anti-MHCII antibody) immunoglobulin G (IgG) secondary antibody (1:1,000; Thermo Fisher Scientific) staining for 2 h at room temperature. Nuclei were detected with 4′,6-diamidino-2-phenylindole (DAPI; 0.1 μg/ml; Sigma-Aldrich). After staining, cells were imaged and analyzed at 531 nm excitation/593 nm emission (Alexa Fluor 555) or at 482 nm excitation/536 nm emission (Alexa Fluor 488; ImageXpress Micro Confocal; Molecular Devices, Sunnyvale, CA, USA). CD68 and MHCII were considered as markers of activated microglia (Hoogland et al., 2015).

The levels of IL-1β, IL-6, and TNF-α in cultured medium pre-treated with 10 μM LM compounds were determined using human Instant IL-1β, IL-6, and TNF-α ELISA kits (Thermo Fisher Scientific), according to the experimental procedures supplied by the manufacturer. The OD at 450 nm was read with Multiskan GO microplate reader (Thermo Fisher Scientific).

### 2.6 Cell viability, lactate dehydrogenase release and ROS production in MPP^+^-treated BE(2)-M17 cells

To examine neuroprotective potential of LM compounds, BE(2)-M17 cells were plated onto 96-well plate [1 × 10^4^/well for cell viability and lactate dehydrogenase (LDH) release assays] or 24-well plate (5 × 10^4^/well for ROS assay) with retinoic acid addition (5 μM) on day 1 to initiate neuronal differentiation. On day 5, after removing retinoic acid, cells were pre-treated with LM compounds (1–10 μM) for 8 h, followed by addition of 0.62 mM MPP^+^ for 40 h. The cell viability was evaluated by MTT assay as described, and the release of LDH in cell culture medium was measured by using LDH cytotoxicity assay kit (Cayman Chemical). The absorbance was read at 490 nm with Multiskan GO microplate reader (Thermo Fisher Scientific).

To detect cellular ROS change on day 7, compounds (10 μM)-treated cells were stained with 2′,7′-dichlorodihydrofluorescein diacetate (DCFH_2_-DA, 10 μM) fluorescein (Thermo Fisher Scientific) and Hoechst 33342 (0.1 μg/ml; Sigma-Aldrich) at 37°C for 30 min. Cell images were captured using FITC [fluorescein isothiocyanate, for 2′,7′-dichlorofluorescein (DCF); 482 nm excitation/536 nm emission] and DAPI filters (ImageXpress Micro Confocal; Molecular Devices).

### 2.7 Tyrosine hydroxylase immunofluorescence staining

BE(2)-M17 cells were seeded in 24-well plate (5 × 10^4^/well) and treated with retinoic acid, LM compounds (10 μM), and MPP^+^ as described. On day 7, cells were fixed (4% PFA), permeabilized (0.1% Triton X-100), blocked (2% BSA), and stained with primary tyrosine hydroxylase (TH; a marker for dopamine-containing neurons; Haavik and Toska, 1998) antibody (#sc-374047; 1:500; Santa Cruz Biotechnology, Santa Cruz, CA, USA) at 4°C overnight, followed by Alexa Fluor 488-donkey anti-mouse IgG secondary antibody (#A-21202; 1:1,000; Thermo Fisher Scientific) at room temperature for 2 h. Nuclei were detected using DAPI (0.1 μg/ml). Dopaminergic differentiation was examined by fluorescence imaging of Alexa Fluor 488 as described.

### 2.8 Caspase activity assays

BE(2)-M17 cells were plated onto six-well (4 × 10^5^/well) and treated with retinoic acid, LM compounds (10 μM), and MPP^+^ as described. Cells were collected on day 7 and lysed by six freeze/thaw cycles. Types of substrates applied were: Ac-Tyr-Val-Ala-Asp-7-amino-4-trifluoromethyl coumarin (YVAD-AFC) for caspase-1, Ac-Val-Glu-Ile-Asp-7-amino-4-trifluoromethyl coumarin (VEID-AFC) for caspase-6 (BioVision, Milpitas, CA, USA), and Ac-Asp-Glu-Val-Asp-7-amido-4-methylcoumarin (DEVD-AMC) for caspase-3 (Sigma-Aldrich). The reaction mixtures were incubated for 1.5 h at 37°C per the manufacturer's instructions. The fluorescence intensity at 400 nm excitation and 505 nm emission (caspase-1 and caspase-6) or 360 nm excitation and 460 nm emission (caspase-3) wavelengths were recorded (BioTek FLx800).

### 2.9 High-content analysis of neurite outgrowth

For automated neuronal morphology analysis, BE(2)-M17 cells were seeded in 24-well plate (5 × 10^4^/well) and treated with retinoic acid, LM compounds (10 μM), and MPP^+^ as described. On day 7, after being fixed and permeated, cells were stained with TUBB3 (neuronal class III β-tubulin) antibody (#802001; 1:1,000; BioLegend, San Diago, CA, USA) at 4°C overnight, followed by Alexa Fluor 555-donkey anti-rabbit (#A-31572) antibody (1:1,000; Thermo Fisher Scientific) at room temperature for 2 h. TUBB3 is a β-tubulin isoform constitutively expressed in neurons and important for maintaining further neurite elongation (Ferreira and Caceres, 1992). After nuclei staining with DAPI, neuronal images were captured at 531 nm excitation and 593 nm emission wavelengths (ImageXpress Micro Confocal; Molecular Devices). Microscopic images were segmented with multi-colored mask to assign each outgrowth to a cell body for quantification of neurite outgrowth. Neurite total length (μm) and numbers of primary neurites originated from neuronal cell body (process) and secondary neurites extended from primary neurites (branch) were analyzed (Neurite Outgrowth Application Module; Molecular Devices). For each sample, around 3,000 cells were analyzed in each of three independent experiments.

### 2.10 Western blot analysis

HMC3 (2 × 10^5^/6-well) or BE(2)-M17 (4 × 10^5^/6-well) cells were lysed in buffer (composition: 50 mM Tris-HCl pH8.0, 2 mM EDTA pH8.0, 150 mM NaCl, 0.5% sodium deoxycholate, 0.1% SDS, 50 mM NaF, 1% NP40) containing the protease and/or phosphatase inhibitor cocktail (Sigma-Aldrich). After sonication, the lysates were centrifuged (12,000 × *g* for 10 min at 4°C) and protein concentrations determined (Bradford protein assay; Bio-Rad, Hercules, CA, USA). Aliquots of protein (20 μg) were separated by electrophoresis using 10%−12% SDS–polyacrylamide gel followed by transfer to a polyvinylidene fluoride (PVDF) membrane. After being blocked, the PVDF membrane was stained with NLRP3 (#15101s), caspase-1 (CASP1; #3866s), iNOS (#20609), IL-1β (#12242), CREB (#9192), BCL2-associated X (BAX; #2772; 1:1,000; Cell Signaling), IL-6 (#ab6672), TNF-α (#ab9739), GCLC (#ab41463), BDNF (#ab108319; 1:1,000; Abcam, Cambridge, UK), NLRP1 (#NBP-1-54899; 1:500; Novus Biologicals, Centennial, CO, USA), NRF2 (#ABE413), NQO1 (#N5288), p-CREB (Ser133; #06-519; 1:1,000; Sigma-Aldrich), PGC-1α (#GTX37356; 1:1,000; GeneTex, Irvine, CA, USA), B-cell lymphoma 2 (BCL2; #IR94-392; 1:1,000; iReal Biotechnology, New Taipei City, Taiwan), or glyceraldehyde-3-phosphate dehydrogenase (GAPDH; #30000002; 1:1,000; MDBio, Taipei, Taiwan) primary antibody at room temperature 2 h or 4°C overnight. The immune complexes were detected using horseradish peroxidase (HRP)-conjugated goat anti-mouse or goat anti-rabbit IgG antibody (1:5,000; GeneTex) and chemiluminescent HRP substrate (Millipore, Burlington, MA, USA). Immunoreactive bands were captured (ImageQuant™ LAS 4000, GE Healthcare, Chicago, IL, USA) and quantified (Multi Gauge V3.0 software, Fujifilm, Tokyo, Japan).

### 2.11 Statistical analysis

For each data set, three independent experiments were performed and data were expressed as the means ± standard deviation (SD). Differences between groups were evaluated by one-way analysis of variance (ANOVA) with a *post hoc* Tukey test (comparing several groups). All *p*-values were two-tailed, with values lower than 0.05 considered being statistically significant.

## 3 Results

### 3.1 Tested LM compounds

**Figure 1:**
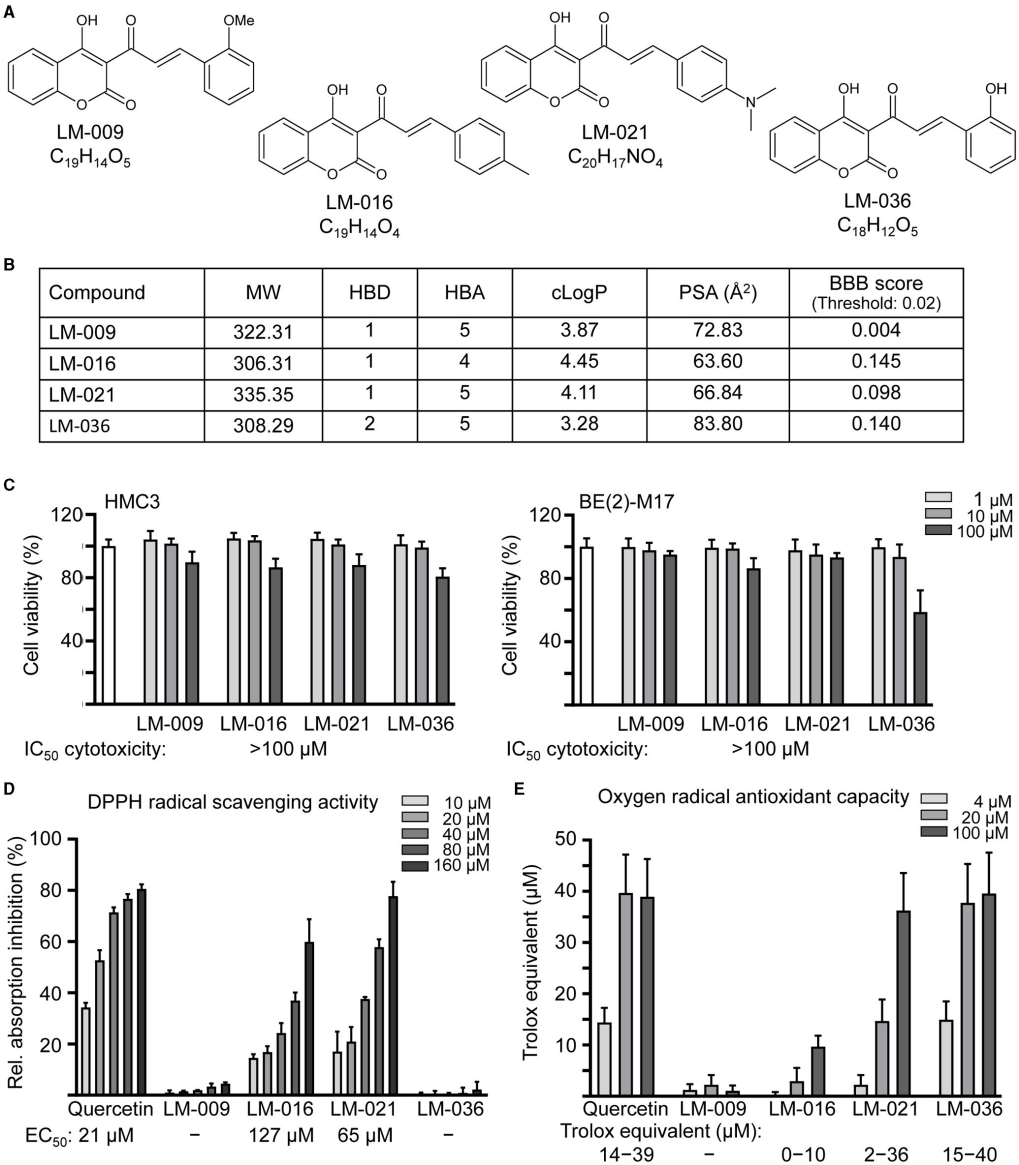
LM compounds. **(A)** Structure and formula of LM-009,−016,−021, and−036. **(B)** Molecular weight (MW), hydrogen bond donor (HBD), hydrogen bond acceptor (HBA), calculated octanol-water partition coefficient (cLogP), polar surface area (PSA), and blood–brain barrier (BBB) permeation score of LM compounds. **(C)** Cytotoxicity of LM compounds against human HMC3 and BE(2)-M17 cells examined by MTT assay. Cells were treated with LM compound (1–100 μM) and cell viability was measured the next day (*n* = 3). To normalize, the relative viability of untreated cells was set at 100%. Shown below were cytotoxicity IC_50_ values. **(D)** DPPH free radical scavenging activity (10–160 μM) and **(E)** oxygen radical absorbance capacity (4–100 μM) of LM compounds and quercetin (as a positive control; *n* = 3). Shown below were EC_50_ or trolox equivalent values.

Four synthetic coumarin-chalcone derivatives were examined (Figure 1A). Oral bioavailability was predicted based on molecular weight (306.31–335.35), hydrogen bond donors (1–2), hydrogen bond acceptors (4–5) and calculated octanol/water partition coefficient (3.28–4.45; Figure 1B). In accordance with calculated PSA (63.60–83.80 Å^2^) and predicted BBB permeation score (0.098–0.145), three of them (LM-016, LM-021, and LM-036) displayed potential of BBB penetration (Figure 1B).

The cytotoxicity of LM compounds (1–100 μM) in HMC3 and BE(2)-M17 cells was examined by MTT assay after 24 h compound treatment. As shown in Figure 1C, all LM compounds had cell viability above 80% in HMC3 cells. In BE(2)-M17 cells, IC_50_ cytotoxicity values were also >100 μM. The results demonstrated the low cytotoxicity of these LM compounds.

To assess the antioxidative properties of LM compounds, DPPH radical scavenging activity and oxygen radical antioxidant capacity were examined. EC_50_ values of DPPH radical scavenging activity for quercetin (positive control), LM-016, and LM-021 were 21, 127, and 65 μM, respectively (Figure 1D). In addition, oxygen radical antioxidant capacity was determined based on the trolox standard curve. At 4–100 μM concentration, quercetin, LM-016, LM-021, and LM-036 had trolox equivalent activity of 14–39, 0–10, 2–36, and 15–40 μM, respectively (Figure 1E). Among the four LM compounds, LM-021 displayed good ability to scavenge both DPPH and oxygen radicals.

### 3.2 Activation of HMC3 microglia and anti-inflammatory potential of LM compounds

**Figure 2:**
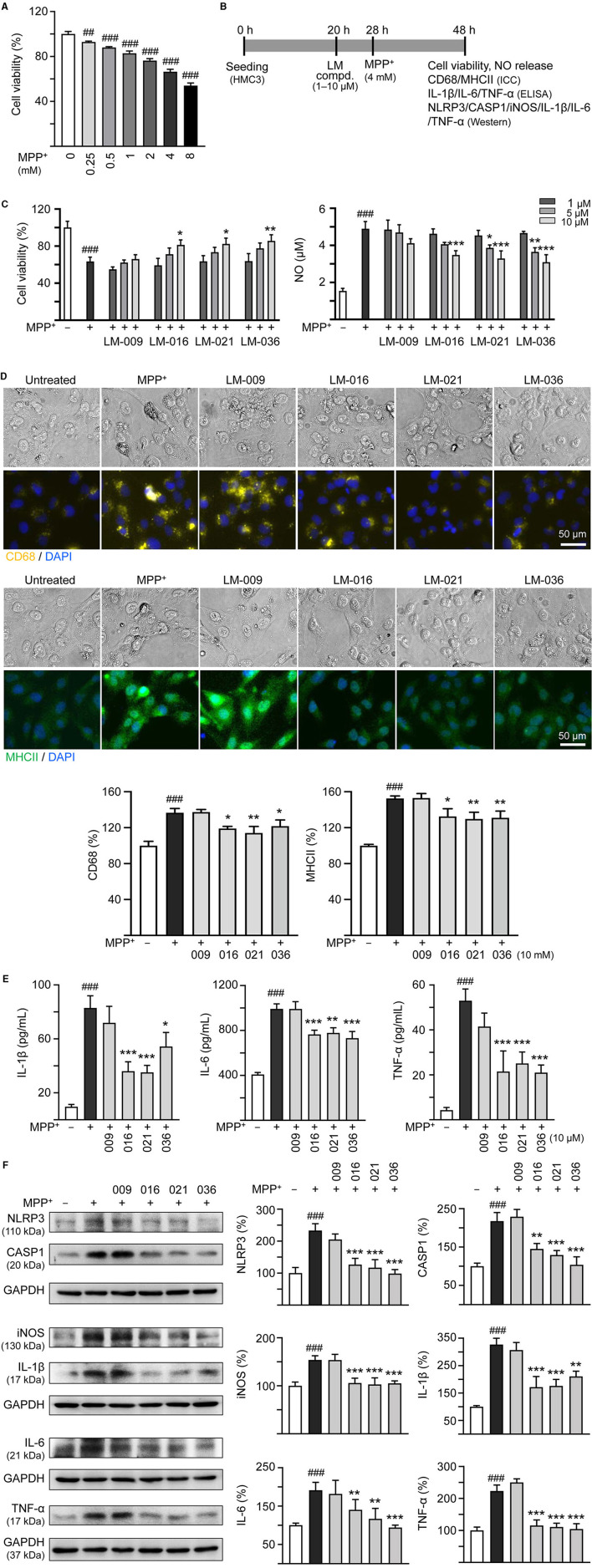
MPP^+^ induced inflammation of HMC3 microglia and anti-inflammatory potential of LM compounds. **(A)** Cytotoxicity of MPP^**+**^ against HMC3 cells by MTT assay. Cells were treated with MPP^**+**^ (0–8 mM) and cell viability was measured the following day (*n* = 3). **(B)** Experimental flow chart of anti-inflammatory test. HMC3 cells were plated on day 1. After 20 h, cells were pre-treated with LM compounds (1–10 μM) for 8 h, followed by MPP^**+**^ (4 mM) treatment for 20 h. On day 3, the HMC3 cells were examined for cell viability and NO release in culture medium **(C)**. In addition, cells with 10 μM compound treatment were examined for CD68 and MHCII expression by ICC staining **(D)**, IL-1β, IL-6, and TNF-α in culture medium by ELISA **(E)**, and NLRP3, CASP1, iNOS, IL-1β, IL-6, and TNF-α expression by Western blotting **(F)** (*n* = 3). The relative cell viability as well as CD68, MHCII, NLRP3, CASP1, iNOS, IL-1β, IL-6, and TNF-α expression levels in MPP^**+**^-untreated cells were normalized as 100%. In CD68 (yellow) and MHCII (green) staining cells, nuclei were counterstained with DAPI (blue). GAPDH was used as a loading control in immunoblotting. *p*-values: comparisons between MPP^+^-untreated and treated cells (^##^*p* < 0.01, ^###^*p* < 0.001), or with and without compound addition (*p < 0.05, **p < 0.01, ***p < 0.001).

To investigate the cytotoxicity of MPP^+^ on human microglial cells, a series of concentrations of MPP^+^ (0–8 mM) were added to HMC3 cells for 20 h. As shown in Figure 2A, MPP^+^ at concentration range of 0.25–8 mM significantly decreased HMC3 cell viability compared to no MPP^+^ treatment (from 100 to 93–54%, *p* = 0.005– <0.001). MPP^+^ at 4 mM concentration was selected to test anti-inflammatory potential of LM compounds. HMC3 cells were pre-treated with LM compounds (1–10 μM) for 8 h before MPP^+^ addition for 20 h (Figure 2B). As shown in Figure 2C, LM-016, LM-021, and LM-036 at 10 μM effectively increased cell viability (from 63 to 81–86%, *p* = 0.045–0.005) and decreased release of NO in cell culture medium (from 4.9 to 3.5–3.1 μM, *p* < 0.001). The observation of reduced expression of CD68 (from 137 to 122–114%, *p* = 0.04–0.002) and MHCII (from 153 to 132–130%, *p* = 0.015–0.006) also demonstrated the anti-inflammation activity of LM-016, LM-021, and LM-036 at 10 μM concentration in MPP^+^-activated HMC3 cells (Figure 2D). The anti-inflammatory potential of these three LM compounds at 10 μM was further supported by the reduced release of IL-1β (from 83 to 54–36 pg/ml, *p* = 0.012– <0.001), IL-6 (from 992 to 779–732 pg/ml, *p* = 0.001– <0.001) and TNF-α (from 53 to 25–21 pg/ml, *p* < 0.001) in cell culture medium (Figure 2E).

Next, we examined the protein levels of NLRP3, CASP1, iNOS, IL-1β, IL-6, and TNF-α in MPP^+^-stimulated HMC3 cells with or without LM compounds treatment. As shown in Figure 2F, MPP^+^ addition increased the expressions of NLRP3 (233%), CASP1 (218%), iNOS (156%), IL-1β (326%), IL-6 (182%), and TNF-α (223%; *p* < 0.001), whereas treatment with LM-016, LM-021, and LM-036 at 10 μM concentration significantly reduced the levels of these proteins involved in inflammasome and inflammatory mediators (NLRP3: 127–99%, CASP1: 145–104%, iNOS: 106–103%, IL-1β: 211–172%, IL-6: 124–90%, TNF-α: 115–105%; *p* = 0.006– <0.001).

### 3.3 Protection of LM compounds against MPP^+^-induced neurotoxicity in BE(2)-M17 cells

**Figure 3:**
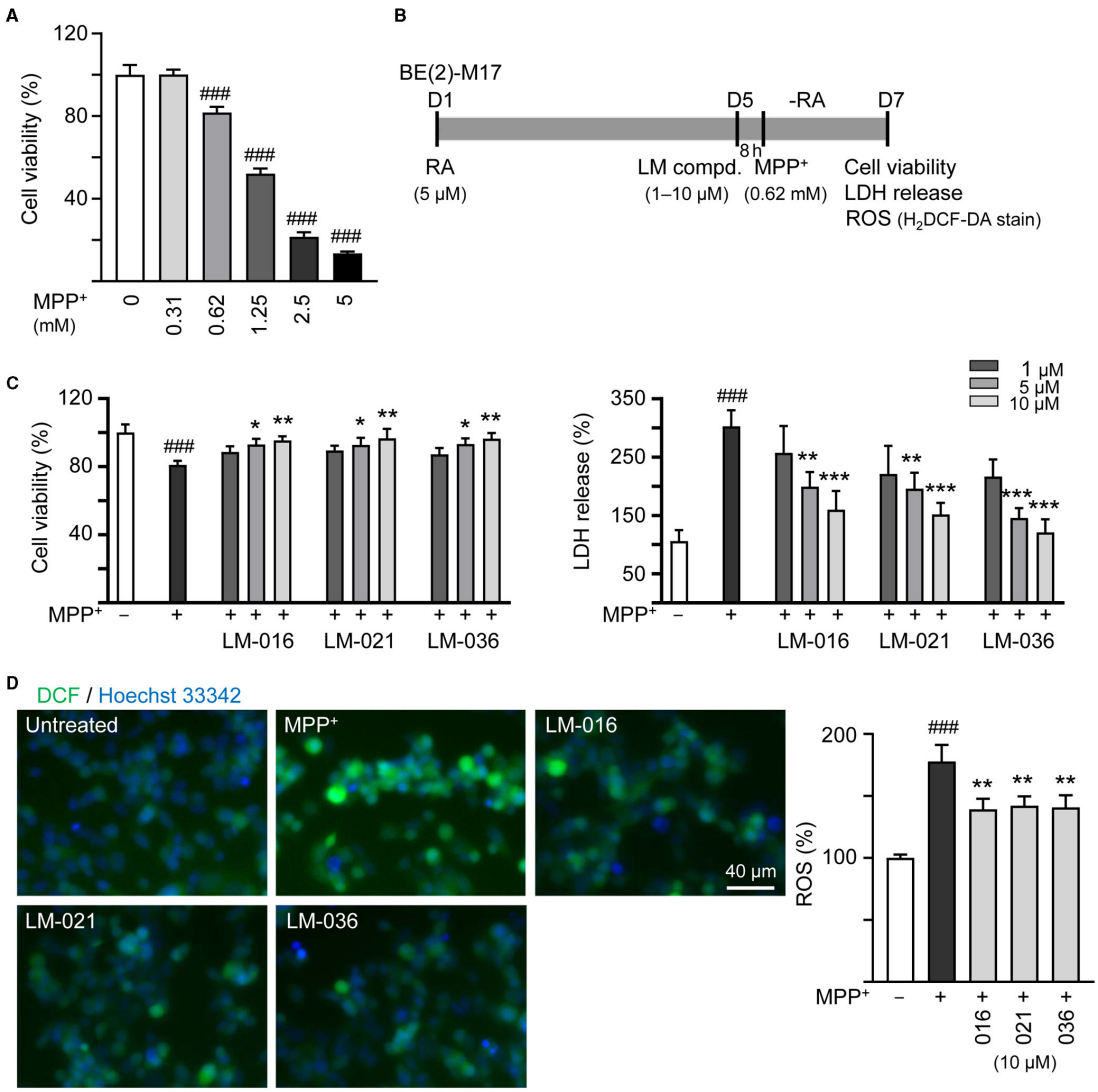
Reduction of cytotoxicity and ROS of LM compounds in MPP^+^-treated BE(2)-M17 cells. **(A)** Cytotoxicity of MPP^**+**^ against BE(2)-M17 cells by MTT assay. Cells were treated with MPP^**+**^ (0–5 mM) and cell viability was measured after 48 h (*n* = 3). The relative cell viability in MPP^**+**^-untreated cells was set at 100%. MPP^**+**^ at 0.62 mM concentration (80% cell viability) was selected for testing anti-inflammatory potential of LM-016, LM-021, and LM-036. **(B)** Experimental flow chart. On day 1, BE(2)-M17 cells were plated in the presence of retinoic acid (RA, 5 μM). On day 5, after removing retinoic acid, cells were treated with LM compounds (1–10 μM) and MPP^**+**^ (0.62 mM). **(C)** cell viability, LDH release, and **(D)** ROS production (H_2_DCF-DA stain) were measured on day 7 (*n* = 3). The relative cell viability, LDH release, and ROS production in MPP^**+**^-untreated cells was set as 100%. Cellular ROS were analyzed by assessing fluorescence of DCF (green) and cell nuclei counterstained with Hoechst 33342 (blue). *p*-values: comparisons between MPP^**+**^-untreated and treated cells (^###^*p* < 0.001), or with and without compound addition (*p < 0.05, **p < 0.01, ***p < 0.001).

MPP^+^ is frequently used to mimic the microenvironment of PD in human neuronal cells (Ye et al., 2017). To investigate the cytotoxicity of MPP^+^ on human BE(2)-M17 cells, increasing concentrations of MPP^+^ (0–5 mM) were added to BE(2)-M17 cells for 20 h. As shown in Figure 3A, MPP^+^ at concentration range of 0.62–5 mM significantly decreased BE(2)-M17 cell viability compared to no MPP^+^ treatment (from 100 to 80–14%, *p* < 0.001). MPP^+^ at 0.62 mM concentration was considered for further experiments. BE(2)-M17 cells were treated with retinoic acid on day 1 to initiate neuronal differentiation. On day 5, after removing retinoic acid, cells were pre-treated with LM compounds (1–10 μM) for 8 h, followed by addition of MPP^+^ for 40 h (Figure 3B). As shown in Figure 3C, LM-016, LM-021, and LM-036 at 10 μM effectively increased cell viability (from 81 to 95–97%, *p* = 0.004–0.002) and decreased the LDH release in cell culture medium (from 302 to 160–121%, *p* < 0.001). The observation of decreased production of ROS (from 178 to 142–139%, *p* = 0.005–0.003) also supported the protective role of LM-016, LM-021, and LM-036 against MPP^+^-induced neurotoxicity in BE(2)-M17 cells (Figure 3D).

### 3.4 TH, caspase-1, caspase-3, caspase-6, and neurite outgrowth assessments in MPP^+^-treated BE(2)-M17 cells

**Figure 4:**
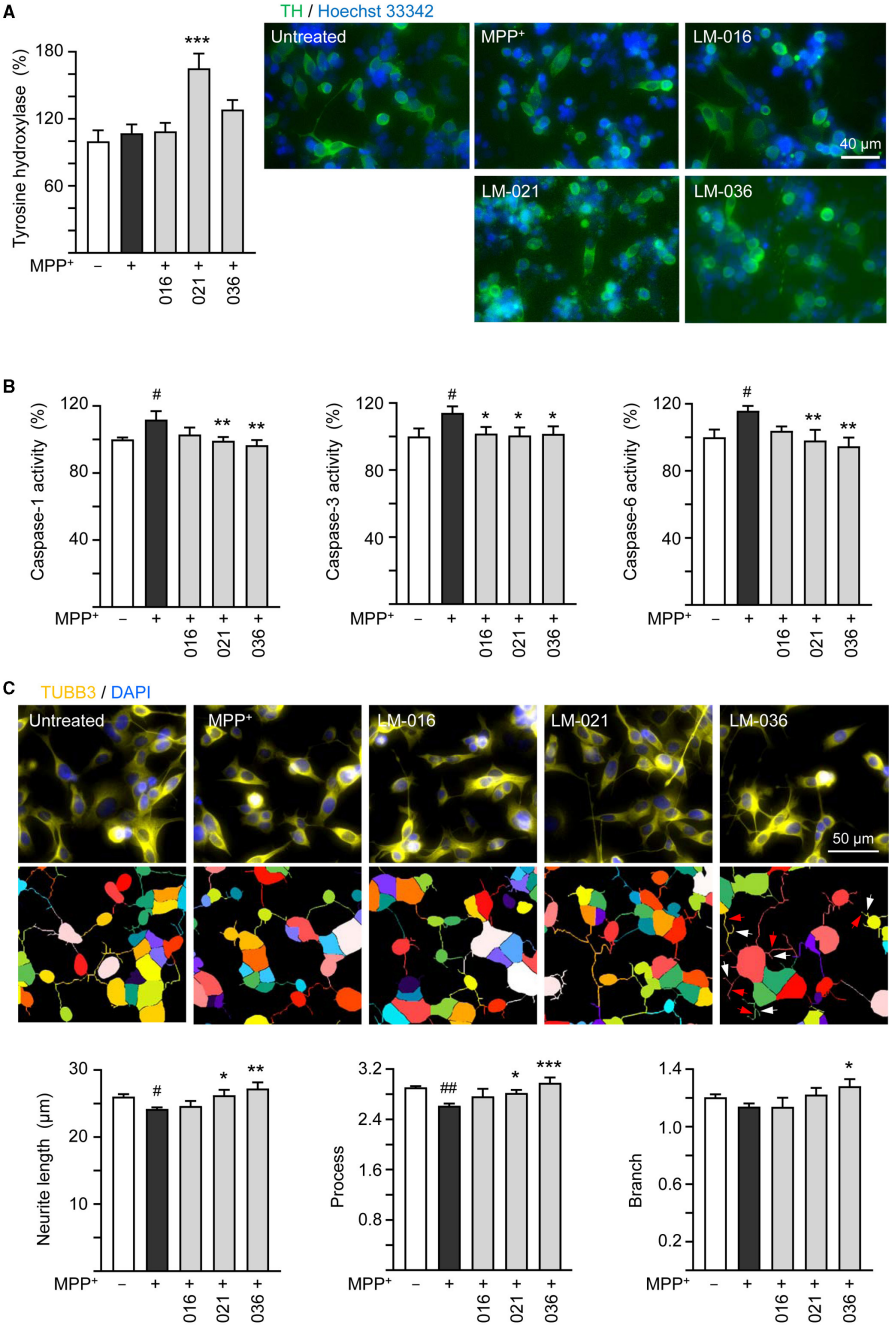
Neuroprotective effects of LM compounds on MPP^+^-treated BE(2)-M17 cells. Retinoic acid-differentiated BE(2)-M17 cells were treated with LM-016, LM-021, or LM-036 (10 μM) and MPP^+^ (0.62 mM) on day 5. On day 7, **(A)** tyrosine hydroxylase (TH, green) expression, **(B)** caspase-1, caspase-3 and caspase-6 activities, as well as **(C)** neurite length, process and branch were measured (*n* = 3). Shown were TUBB3-stained images and images of cells outlined with multi-colored mask to assign each outgrowth to a cell body for neurite outgrowth quantification. Processes and branches in LM-036-treated cells were marked with red and white arrows, respectively. To normalize, TH expression and caspase activities in MPP^+^-untreated cells were set at 100%. *p*-values: comparisons between MPP^+^-untreated and treated cells (^#^*p* < 0.05, ^##^*p* < 0.01), or with and without compound addition (*p < 0.05, **p < 0.01, ***p < 0.001).

It has been reported that MPP^+^ administration decreases the TH protein expression in SH-SY5Y cells (Zhu et al., 2019; Kang et al., 2021; Rani et al., 2023). To further analyze MPP^+^-induced cytotoxicity and neuroprotective impact of LM compounds in BE(2)-M17 cells, we assessed the expression of TH. As described in Figure 3B, after retinoic acid-induced neuronal differentiation for 5 days, BE(2)-M17 cells were treated with MPP^+^ (0.62 mM) for 40 h, and dopaminergic characteristic of BE(2)-M17 cells was examined using immunofluorescence staining of TH. MPP^+^ treatment did not significantly affect TH immunofluorescence compared to the untreated control (107 vs. 100%, *p* > 0.05). Among the three examined LM compounds, LM-021 significantly increased TH expression in MPP^+^-treated BE(2)-M17 cells (from 107 to 165%, *p* < 0.001), whereas the TH expression promoted by LM-036 was not significant (128%, *p* = 0.123; Figure 4A).

Caspase-1 and caspase-3 have been shown to play a role in the course of injury induced by MPP^+^ in PC12 cells (Xu et al., 2015). In addition, caspase-1 activates caspase-6 in human primary central nervous system (CNS) neuronal cell cultures (Guo et al., 2006) and active caspase-6 is associated with axonal degeneration (Uribe et al., 2012). Consequently, caspase-1, caspase-3, and caspase-6 activities in these LM compounds-treated cells were examined. The addition of MPP^+^ significantly raised activity of caspase-1 (112%, *p* = 0.013), caspase-3 (114%, *p* = 0.02), and caspase-6 (116%, *p* = 0.012). In comparison to no treatment, LM-021 and LM-036 (10 μM) treatments reduced the activity of caspase-1 (99%−97%, *p* = 0.008–0.002), caspase-3 (101%, *p* = 0.037–0.026), and caspase-6 (98%−95%, *p* = 0.006–0.002; Figure 4B). For LM-016, only the reduction of caspase-3 activity was significant (102%, *p* = 0.041). Consistently, addition of MPP^+^ significantly decreased total length of neurite (from 26.0 to 24.2 μm, *p* = 0.048) and number of process (from 2.9 to 2.6, *p* = 0.004); LM-021 and LM-036 (10 μM) treatment successfully rescued the decrease of neurite length (from 24.2 to 26.2–27.2 μm; *p* = 0.029–0.002) and process (from 2.6 to 2.8–3.0; *p* = 0.041– <0.001; Figure 4C). Nevertheless, the reduction of branch after MPP^+^ treatment was not significant (from 1.2 to 1.1, *p* > 0.05).

### 3.5 Protection of MPP^+^-induced neurotoxicity via regulating neuroinflammatory, antioxidative and neuroprotective pathways

**Figure 5:**
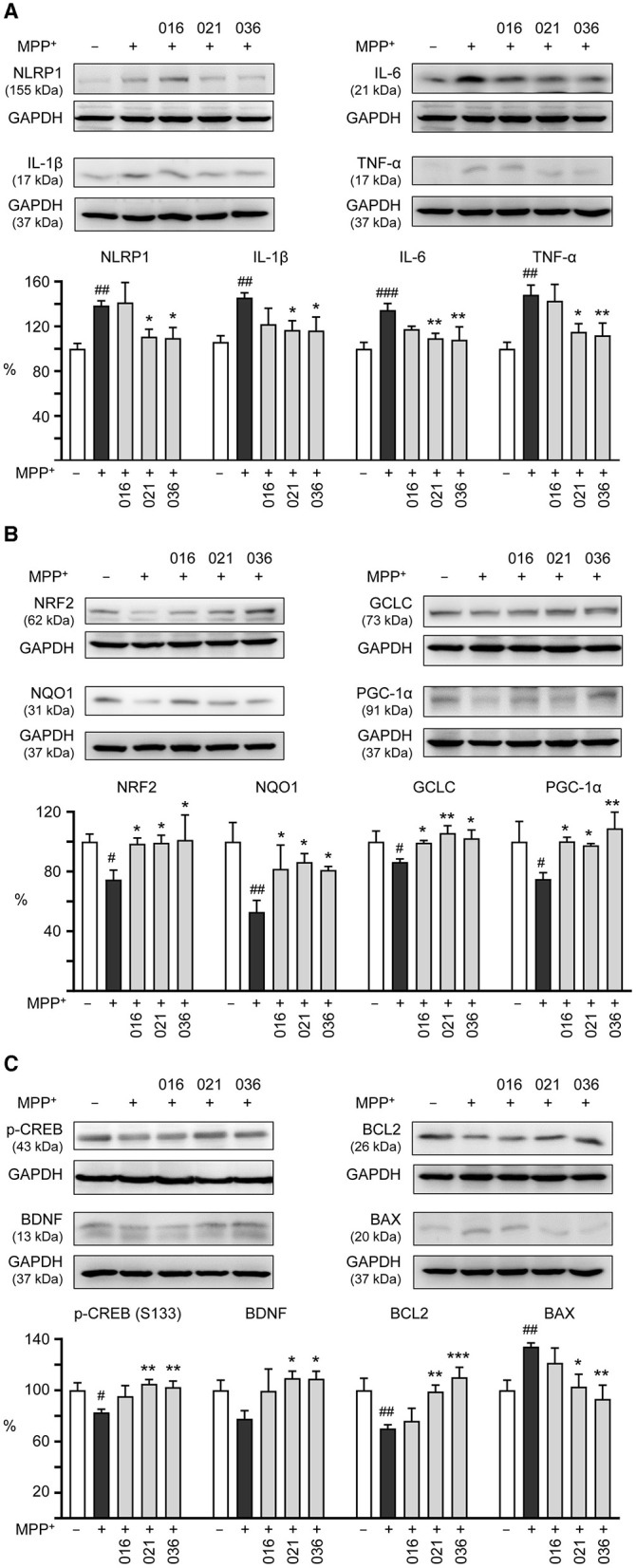
Neuroinflammatory, antioxidative, and neuroprotective effects of LM compounds on MPP^+^-treated BE(2)-M17 cells. Cells were treated as Figure 4 described. **(A)** Neuroinflammatory NLRP1, IL-1β, IL-6, and TNF-α, **(B)** antioxidative NRF2, NQO1, GCLC, and PGC-1α, and **(C)** neuroprotective CREB, BDNF, and BCL2, and pro-apoptotic BAX were examined (*n* = 3). GAPDH was included as a loading control. To normalize, the relative protein levels in MPP^+^-untreated cells were set at 100%. *p*-values: comparisons between MPP^+^-untreated and treated cells (^#^*p* < 0.05, ^##^*p* < 0.01, ^###^*p* < 0.001), or with and without compound addition (*p < 0.05, **p < 0.01, ***p < 0.001).

We then examined if LM compounds downregulate inflammation-related NLRP1, IL-1β, IL-6, and TNF-α gene expression in MPP^+^-treated BE(2)-M17 cells. As shown in Figure 5A, treatment with LM-021 or LM-036 reduced the NLRP1 (from 138 to 111–110%, *p* = 0.041–0.032), IL-1β (from 146 to 117–116%, *p* = 0.028–0.026), IL-6 (from 134 to 109–108%, *p* = 0.007–0.005), and TNF-α (from 148 to 115–112%, *p* = 0.014–0.008) levels. In addition, cellular redox signaling including NRF2, NQO1, GCLC, and PGC-1α in MPP^+^-treated BE(2)-M17 cells was examined. Treatment with LM-016, LM-021, and LM-036 elevated the NRF2 (from 75 to 99–101%, *p* = 0.045–0.026), NQO1 (from 53 to 81–86%, *p* = 0.043–0.016), GCLC (from 86 to 99–106%, *p* = 0.048–0.004), and PGC-1α (from 75 to 98–109%, *p* = 0.042–0.006) levels (Figure 5B). Moreover, LM-021 and LM-036 treatment up-regulated p-CREB (Ser133; from 83 to 102–105%, *p* = 0.008–0.003) and downstream neurotrophic factor BDNF (from 78 to 109–110%, *p* = 0.017–0.016) and anti-apoptotic molecule BCL2 (from 70 to 99–110%, *p* = 0.006– <0.001), accompanied by decreased pro-apoptotic protein BAX (from 134 to 103–93%, *p* = 0.013–0.002; Figure 5C).

## 4 Discussion

Microglia, the main neuroimmune cells in the CNS, are key mediators of neuronal damage in neurodegenerative diseases (Block et al., 2007). Chronic neuroinflammation is one of the hallmarks of PD pathophysiology and inflammatory processes have been suggested as promising interventional targets for PD (Wang et al., 2015). In this study, we demonstrate that coumarin-chalcone derivatives LM-016, LM-021, and LM-036 alleviate MPP^+^-induced production of inflammatory mediators in HMC3 microglia (Figure 2). By down-regulating neuroinflammatory and up-regulating antioxidative and neuroprotective pathways, LM-021 and LM-036 increase cell viability, ameliorate cellular ROS production, and promote neurite outgrowth in MPP^+^-treated BE(2)-M17 cells (Figures 3–5). The study results strengthen the roles of oxidative stress and neuroinflammation in PD pathogenesis, and suggest the potential of LM-021 and LM-036 for the treatment of PD.

Activation of microglia triggers a cascade of neuroinflammatory processes that are thought to play an important role in neurodegeneration (Hickman et al., 2018). The microglial NLRP3 activation leads to caspase-1-mediated proteolytic activation of the IL-1β to induce inflammation and cell death, and pharmacological inhibition of NLRP3 activation may be beneficial to mitigate the progression of inflammatory diseases (Mangan et al., 2018). A selective NLRP3 inflammasome inhibitor MCC950 (a sulfonylurea derivative) attenuates behavioral deficits and neuroinflammation in MPTP-induced mouse model of PD (Huang S. et al., 2021). Moreover, synthetic dl-3-n-butylphthalide (dl-NBP) suppresses NLRP3 inflammasome activation and protects dopaminergic neurons in MPTP-induced mouse and 6-hydroxydopamine (6-OHDA)-induced SH-SY5Y cell models (Que et al., 2021). Currently there are no approved drugs targeting NLRP3 for clinical use. Chalcone has been suggested as a potential scaffold for NLRP3 inflammasome inhibitors (Thapa et al., 2023). In the present study, the coumarin-chalcone derivatives LM-016, LM-021, and LM-036 inhibited the expression of NLRP3 and CASP1 to reduce the release of NO, IL-1β, IL-6, and TNF-α in MPP^+^-induced human microglia (Figure 2), and this suppression may be due to the modifications of NLRP3 by α, β-unsaturated bonds and electron withdrawing groups of these derivatives (El-Sharkawy et al., 2020). Although with α, β-unsaturated carbonyl Michael acceptor group, the steric hindrance of ortho-methoxy group on B-ring of LM-009 (Figure 1) may affect the covalent bond formation with NLRP3, thus disrupting the functional inhibition. As the predicted BBB score may not reflect physiological conditions, LM-009 with BBB score <0.02 threshold was also included for comparison in MPP^+^-induced human microglia.

In previous studies, we showed that LM-021 and LM-036 exert neuroprotection through upregulating CREB and its downstream BDNF and BCL2 in Aβ-GFP and ΔK280 Tau_RD_-DsRed folding reporter SH-SY5Y cells (Chiu et al., 2021; Huang C. C. et al., 2021; Weng et al., 2023). LM-021 increases CREB-mediated gene expression through protein kinase A (PKA), Ca^2+^/calmodulin-dependent protein kinase II (CaMKII), and extracellular signal-regulated kinase (ERK) (Chiu et al., 2021), whereas LM-036 activates tropomyosin-related kinase-B (TRKB)-mediated ERK and AKT serine/threonine kinase 1 (AKT) signaling (Huang C. C. et al., 2021; Weng et al., 2023). In the current study, LM-021 and LM-036 promoted cell survival and exhibited neuroprotective efficacy in MPP^+^-induced BE(2)-M17 cells by suppressing NLRP1-associated inflammation, activating NRF2-induced expression of antioxidants and PGC-1α, and enhancing CREB-promoted expression of neuroprotective BDNF and BCL2 (Figure 5). It is worthwhile to further investigate if TRKB, PKA, CaMKII, ERK, and/or AKT signaling pathways are also activated by LM-021 and LM-036 in MPP^+^-treated BE(2)-M17 cells. As a link between Tau and Aβ deposition and cognitive decline in PD (Athauda and Foltynie, 2015), it is also worthwhile to evaluate the potential efficiency of LM-021 and LM-036 in treatment of cognitive impairments in PD.

NLRP1, the first reported protein shown to form an inflammasome (Martinon et al., 2002), is expressed in glandular epithelial structures such as stomach, gut, lung, and in neurons and testis (Kummer et al., 2007). When activated, formation of NLRP1 inflammasome leads to the production of proinflammatory cytokines to augment neurological disorders, cardio-pulmonary diseases and cancer through promoting inflammation (Tupik et al., 2020). Given the growing recognition and awareness of the role of NLRP1 in human diseases, pharmacological targeting of NLRP1 has provided a potential therapeutic to treat NLRP1-associated diseases. In human cells, dipeptidyl peptidase 9 (DPP9) functions as an endogenous inhibitor of NLRP1 inflammasome via both peptidase activity and function-to-find domain binding (Zhong et al., 2018). In addition, ubiquitously expressed endogenous thioredoxin has been identified as a binder of NLRP1 and a suppressor of the NLRP1 inflammasome (Zhang et al., 2023). Recently, Docherty et al. (2023) reported that a sulfonylurea compound ADS032 acts as a dual NLRP1 and NLRP3 inhibitor to reduce secretion and maturation of IL-1β in human-derived macrophages and bronchial epithelial cells in response to the activation of NLRP1 and NLRP3. In our study, both coumarin-chalcone compounds LM-021 and LM-036 function as dual NLRP1 and NLRP3 inhibitors to suppress MPP^+^-induced inflammation in human microglia HMC3 and neuroblastoma BE(2)-M17 cells. The mechanisms underlying LM-021- and LM-036-mediated regulation of NLRP1 and NLRP3 remain to be elucidated.

There is currently no disease-modifying treatment for PD. Potential strategies targeting oxidation and/or inflammation using MPP^+^/MPTP-induced PD models have been explored to halt or decelerate the disease progression. For example, quercetin, a flavonoid with multiple pharmacological activities, inhibits ferroptosis by activating NRF2 expression to protect against MPP^+^/MPTP-induced dopaminergic neuron death (Lin et al., 2022). Baicalein, a bioactive flavone widely used in nutraceuticals and pharmaceuticals, prevents MPP^+^/MPTP-induced neurotoxicity via suppressing oxidative stress and inhibiting ERK activation (Song et al., 2021). In addition, baicalin also protects against MPP^+^-and MPTP-induced dopaminergic neuron loss and improve motor performance by upregulating NRF2 and inhibiting NLRP3 (Huang et al., 2024). Hyperoside, a flavonol glycoside with a broad spectrum of biological activities, attenuates MPP^+^/MPTP-induced injuries of dopaminergic neurons through reducing NO, H_2_O_2_, and malondialdehyde, as well as the mitochondrial damage (Xu et al., 2023). In dopaminergic neurons derived from α-synuclein-expressing SH-SY5Y cells, both NLRP1 and NLRP3 inflammasomes are activated to promote the release of IL-1β (Chen et al., 2021). In the present study, LM-021 and LM-036 exert neuroprotective effects by suppressing neuroinflammation, oxidative stress, and enhancing NRF2. It is of noted that LM-021 and LM-036 demonstrate good bioavailability and BBB penetration potential in *in vivo* pharmacokinetic assessment (Chiu et al., 2021) or parallel artificial membrane permeability assay (Huang C. C. et al., 2021), further enhancing their potential translation to clinical practice. Future studies in animal models of PD will be necessary to validate their potential in treating PD before moving toward to clinical trials.

TH enzyme in the CNS is responsible for catalyzing the conversion of tyrosine to L-3,4-dihydroxyphenylalanine (L-DOPA), a precursor for dopamine. Reduction of TH expression results in diminished dopamine synthesis; therefore, dysregulation of TH activity contributes to the development of PD (Zhu et al., 2012). MPP^+^ treatment (15 μM) for 48 h reduces the expression of TH mRNA in cultured dopaminergic neurons (Beck et al., 1991). In MPP^+^ (100 μM)-treated pheochromocytoma PC12 cells, TH mRNA expression transiently increased (2–24 h) and then gradually decreased (2–6 days) below the control (Itano et al., 1995). In addition, MPP^+^ (0.5–3 mM) treatments for 24 h decrease the TH protein expression in SH-SY5Y cells (Zhu et al., 2019; Kang et al., 2021; Rani et al., 2023). In our study, MPP^+^ (0.62 mM) treatment of neuronally differentiated BE(2)-M17 cells for 40 h did not decrease TH expression (Figure 4A). The cytotoxicity of 0.62 mM MPP^+^ on differentiated BE(2)-M17 cells may be modest so that the TH is relatively preserved. As MPP^+^ treatment timing, concentration, and duration may affect TH expression, further experimental research is required to elucidate the impact of MPP^+^ on TH expression in BE(2)-M17 cells. Human TH promoter contains a TATA box and consensus binding sequences for basal and dopaminergic neuron-specific transcription factors (Kim et al., 2003). Although the increased neuronal survival may increase TH expression, the TH immunofluorescence may be increased by LM-021 and LM-036 through transcriptional regulation of TH expression.

Although our study has shown the neuroprotective effects of the test compounds on MPP^+^- induced cell models, it is arguable that toxin-based animal models may not faithfully reflect the human PD, because human PD is a slowly progressive neurodegeneration, but severe phenotype and extensive pathology can be induced by MPTP in just a few days (Athauda and Foltynie, 2015; Huenchuguala and Segura-Aguilar, 2024a). Studies of single-neuron neurodegeneration as a degenerative model for PD propose that an endogenous neurotoxin, aminochrome, affects a single neuron and triggers the neurodegenerative process, which can be protected by DT-diaphorase (NQO1) and glutathione transferase M2-2, the downstream enzymes of NRF2 (Huenchuguala and Segura-Aguilar, 2024a,b). Our study has also shown that NRF2 and its downstream antioxidants (GCLC and NQO1) are upregulated by the test compounds to promote cell survival and neurite outgrowth (Figure 5B). Certainly, these results should be validated using a model more mimicking human PD in the future.

## 5 Conclusion

**Figure 6:**
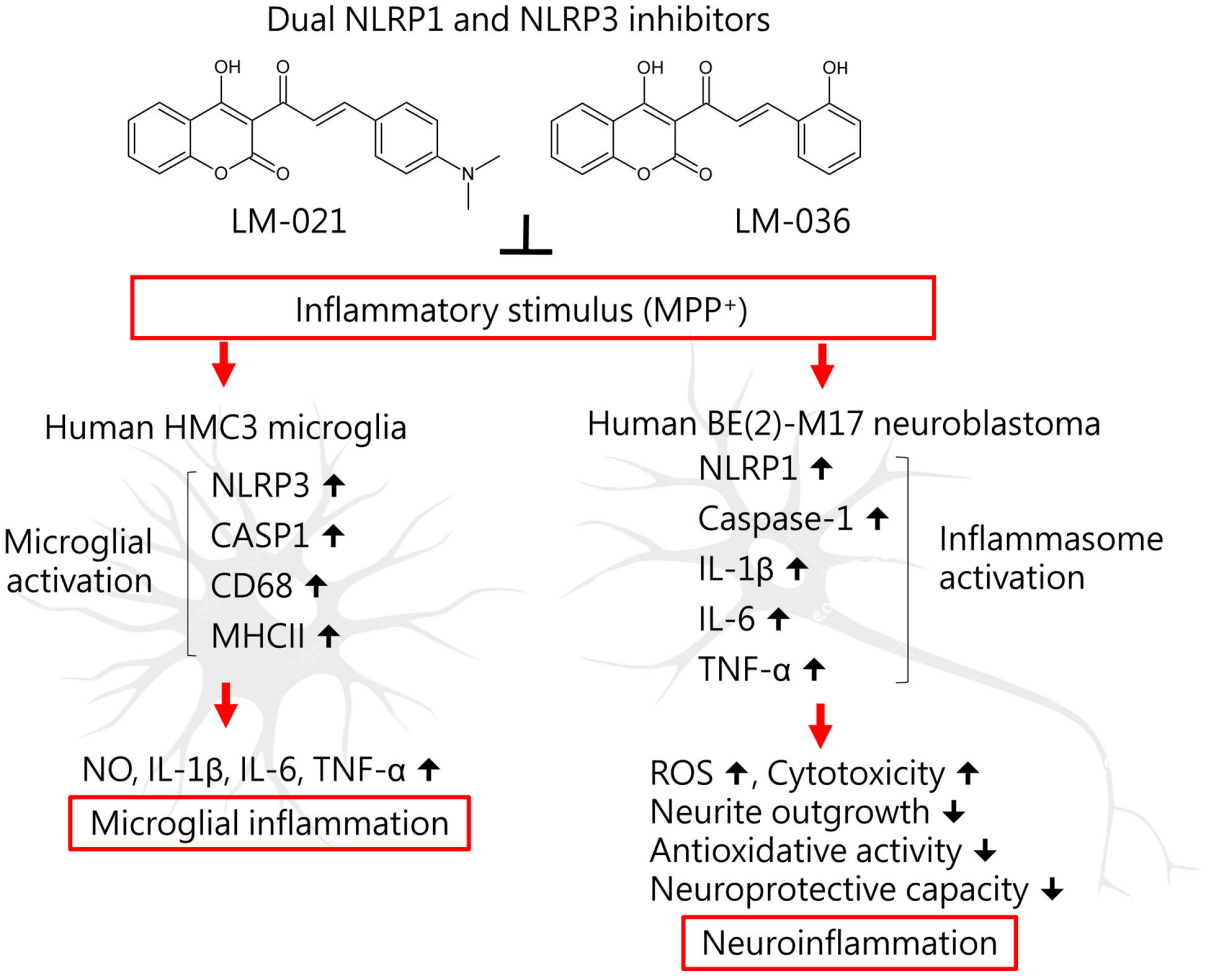
Graphical summary of coumarin-chalcone derivatives LM-021 and LM-036 as dual NLRP1 and NLRP3 inhibitors in MPP^+^-induced HMC3 and BE(2)-M17 cell models of PD.

This study shows anti-inflammatory and neuroprotective effects of coumarin-chalcone derivatives LM-021 and LM-036 in MPP^+^-induced HMC3 and BE(2)-M17 cell models of PD (Figure 6). The two compounds inhibit NLRP3 and CASP1 to attenuate MPP^+^-induced HMC3 microglial activation, leading to decreased production of NO, IL-1β, IL-6, and TNF-α, as well as suppression of CD68 and MHCII expression. In MPP^+^-treated BE(2)-M17 cells, the administration of LM-021 and LM-036 effectively reduced activity of caspase-1, caspase-3, caspase-6, and cytotoxicity, while diminishing ROS level and facilitating neurite outgrowth. Neuroprotective effects of LM-021 and LM-036 may result from the reduction of inflammatory NLRP1, IL-1β, IL-6, and TNF-α, and augmentation of antioxidative NRF2, NQO1, GCLC, and PGC-1α, as well as neuroprotective CREB, BDNF, and BCL2. Overall, the encouraging findings indicate that LM-021 and LM-036 hold great potential as viable options for the treatment of PD.

## Data Availability

The original contributions presented in the study are included in the article/supplementary material, further inquiries can be directed to the corresponding authors.
